# Influence of Highly Accessible Urban Food Environment on Weight Management: A Qualitative Study in Seoul

**DOI:** 10.3390/ijerph15040755

**Published:** 2018-04-14

**Authors:** Nan-He Yoon, Seunghyun Yoo, Soonman Kwon

**Affiliations:** 1 Department of Health Administration, Hanyang Cyber University, Wangsimni-ro 220, Seongdong Gu, Seoul 04763, Korea; yoon_nh@daum.net; 2 Department of Public Health Science, Graduate School of Public Health, Seoul National University, 1 Gwanak-ro, Gwanak-gu, Seoul 08826, Korea; kwons@snu.ac.kr (S.K.); 3 Institute of Health and Environment, Seoul National University, 1 Gwanak-ro, Gwanak-gu, Seoul 08826, Korea

**Keywords:** food environment, obesity, weight management, urban health, photo elicitation interview, qualitative research

## Abstract

We explored the characteristics of the food environment and its influence on weight management in Seoul, Korea. Photo elicitation interviews were conducted with 73 participants who took three photographs per topic related to their food environment and discussed these photographs in groups. Through thematic analysis, we identified four themes concerning participants’ perceptions of the food environment and weight management: (1) “convenience comes first,” (2) “tempting food environment,” (3) “alcohol and *anju*,” and (4) “burden of individual effort to manage weight.” A systematic change toward an environment supportive of healthy eating and weight management is recommended.

## 1. Introduction

Obesity is a major global health challenge that contributes to the prevalence and mortality of chronic diseases [1]. While obesity is considered the result of complex interactions and pathways among diverse factors at the individual and environmental levels, the built environment is a strong determinant of weight management, being a source of both opportunities for and barriers to healthy eating [2,3].

Community built environments are considered to have a salient influence on individual dietary behaviors contributing to weight management [4,5], making it a primary focus of both researchers and policymakers [6,7]. The food environment could be either an enabler or a barrier to healthy eating depending on the food offered in that environment [8,9]. Research so far has focused mostly on the accessibility and availability of either healthy or unhealthy food, which is often measured in terms of density or proximity of food retailers, including various types of restaurants, takeout businesses, supermarkets, and convenience stores [5,10]. Most studies, barring some inconsistencies, have shown that quality of diet is positively associated with accessibility to fresh food outlets and negatively associated with closeness to fast food joints or convenience stores [11,12]. In the association between the density of food retailer types and prevalence of obesity among US adults, obesity prevalence was found to increase as the density of supercenters and convenience stores increased; however, it decreased as the density of grocery stores and specialized food stores selling mostly fresh food items increased [13,14].

The influence of the built environment on diet and obesity has been highlighted in North America, Europe, and Australia [15,16,17], but it remains unclear whether the same influence is found in other countries [18]. Urban physical settings may have a similar mix of retail food outlets, but the way people are exposed to and perceive their built food environment varies by city, leading to different influences on eating behaviors [19,20].

Prevalence of obesity—based on a body mass index (BMI) of ≥25, which is the World Health Organization (WHO) standard of obesity for Asians [21,22]—in Seoul is about 25%. Additionally, about 65% of the entire population of Seoul reported having made efforts to lose weight in the past year [23]. Nevertheless, the prevalence of obesity in Seoul has increased continuously, showing significant differences in obesity prevalence based on sociocultural status. Therefore, obesity prevention and weight management are important public health issues in Seoul [24].

Seoul, the capital of South Korea, has a high population density and is characterized by a high degree of mixed land use, as well as an extensive public transportation system. Seoul’s modal split of transit has continued to increase since it surpassed 60% in 2005, reaching 65.8% in 2015 [25]; even higher than New York City’s, which was 56.5% in 2015 [26]. More than 60% of Seoul citizens commute using public transportation [25], which involves walking to and from bus stops or subway stations. Mixed land use in Seoul’s districts allows various food retailers to be in proximity to residential areas and commuting routes. For example, commuters in Seoul encounter various food retailers, such as food stalls, takeout stores, restaurants, and bars, within walking distance of their homes and workplaces. Seoul is a city experiencing rapid change, particularly in its physical and cultural food environments. Changes in food-related service modes, store types, cuisines, packaging, and social norms are assumed to interact with food purchasing and eating behaviors. Seoul is further fraught with public health issues related to weight management and obesity.

Traditional Korean cuisine is characterized by a main dish with rice and a number of side dishes with various ingredients. Aside from items accompanying main dishes, such as steamed rice, soup, and condiments, there are typically three to five side dishes [27]. The traditional Korean cuisine is generally considered a healthy diet, characterized by fresh vegetables and low-fat foods [28]. However, its preparation is now regarded as an intensive time-consuming effort, which makes people with busy urban lives reluctant to cook. Meanwhile, the common diet in Korea has become more Westernized due to changes in lifestyle and the environment [29]. In Korea, eating is perceived not just as food intake but as an important social and communal activity; the Korean word for “family” contains the meaning of “those who eat together” [30]. Interest in eating has recently increased with the changing cultural environment. More than 30 regular TV programs on eating or cooking are broadcast per year, and these are watched by more than 95% of Koreans [31]. The frequency of searching about food or cooking online has also increased more than 150% in the last three years [32].

Considering the above, this study aimed to explore how the characteristics of Seoul’s urban environment, particularly in relation to food and eating lifestyles, influence residents’ weight management. By conducting qualitative research using photo elicitation interviews, we focused on the context of the participants’ experiences and perceptions of urban food environments.

## 2. Materials and Methods

We conducted photo elicitation interviews with 73 adults aged 20–50 years in Seoul from November 2015 to May 2016 to identify which characteristics of the perceived urban environment influence weight management. Photo elicitation interviews have been used to capture participants’ perceptions and experiences using visual data [33]. In a photo elicitation interview, participants take photographs that represent their views and opinions on topics of interest and then discuss their thoughts on the photographs taken [34]. This process causes participants to think more deeply about the topic and even come up with new suggestions [35].

Two of Seoul’s 25 administrative districts were selected for this study based on their prevalence of obesity in the past 5 years. District A had the highest prevalence of obesity and District B the lowest. Each district has about 500,000 residents, and both have mixes of residential and commercial areas. A snowball sampling method was used to recruit participants in each district from local public health centers, community groups for hobbies and cultural activities, and tenants of apartment complexes, to name a few. The inclusion criteria for participants were as follows: those who had lived for longer than 1 year in the respective district at the time of recruitment and those with recent experience of weight control; more specifically, those who had been diagnosed as overweight or obese within the 2 years prior to the study and had since attempted weight control.

In qualitative research processes using photos, 7–10 participants are recommended ideally for a group discussion [36,37]. Following this standard, groups of participants were formed according to gender and age group (20s–30s and 40s–50s) in each district. In addition to gender and age, participants were varied in terms of employment and family status. Among the 73 participants, 16 men in 4 small groups and 23 women in 6 groups were from District A, while 12 men and 22 women in 5 groups each were from District B.

Each group met twice in this study. The first group meeting was conducted to explain the goals, process, and ethics of the study to the participants and to obtain their informed consent. All the recruited participants agreed to take 3 photographs for each of the following topics: (1) their meaning of obesity prevention and management, (2) factors facilitating their behaviors toward obesity prevention and weight management, (3) barriers influencing their behaviors toward obesity prevention and weight management, and (4) suggestions for developing environments that support obesity prevention and management.

Two weeks after the first group meeting, 12 sets of photographs and short statements about the photographs were collected from the participants. The short statement (about half a page per photograph) was guided by 3 questions adapted from the SHOWeD [38] and PHOTO [39] questions, as below (Box 1). These 3 questions were used to define the meanings, contextualize, and promote storytelling regarding the participants’ photographs, thus leading to group discussions [40].

Box 1 **Questions for short statement per each photograph.**

**Original SHOWeD Questions [38]:**

1) What do you **S**ee here?
2) What’s really **H**appening here?
3) How does this relate to **O**ur lives?
4) **W**hy does this problem or this strength exist?
5) What can we **D**o about this?

**Original PHOTO Questions [39]:**

1) Describe your **P**icture.
2) What is **H**appening in your picture?
3) Why did you take a picture **O**f this?
4) What does this picture **T**ell us about your life?
5) How can this picture provide **O**pportunities for us to improve life?

**Guides for photograph statement we provided:**

1) What does this picture mean?
2) How does this picture relate to your efforts for obesity prevention and management?
3) What can we do about this?

A total of 876 sets of photographs and statements (12 pairs of photographs and accompanying statements × 73 participants) were analyzed to identify keywords and common viewpoints from the participants. About 4–5 keywords emerged per topic. After obtaining the results of the initial analysis of the photographs and short statements, we had participants meet again in 20 small groups to share the results of the initial analysis, discuss the results further, and generate suggestions on community environment improvement for obesity prevention and weight management as a group. To enhance the trustworthiness of qualitative studies, the importance of member checking in the analysis process has been emphasized [41,42,43,44]. In particular, the participatory analysis process has been emphasized for qualitative research methods using photographs. For contextual understanding, the research should be complemented, developed, and deepened through repetitive work, not one-time work [36,40,45]. In the group meetings, the photographs and analysis results were shared with the participants, who further discussed them to reach agreement for the study’s findings.

With the participants’ consent, each group discussion was audio recorded and transcribed verbatim for further analysis. All collected data, including photographs, statements, and transcripts were analyzed using thematic analysis [46]. We derived 163 codes through an initial open-coding process, which involved separating, grouping, regrouping, and relinking data to integrate meanings and explanations [47]. Each code was data driven based on the meaningful content of the participants’ experiences and perceptions of weight management and its influencing factors. We then organized similarly coded data and clustered them into 12 categories and 23 subcategories that shared the similar characteristics. By repeating the process of categorizing and refining the categories [48], 4 subtly and implicitly descriptive themes were developed to explain the overarching factors influencing weight management: (1) “convenience comes first,” (2) “tempting food environment,” (3) “alcohol and *anju*,” and (4) “burden of individual effort to manage weight.”

Of the 163 codes, 41 codes including busy daily life, time pressure, fast foods, instant foods, and single households formed 6 subcategories, and then 3 categories related to preference for convenient and quick ways to eat, leading to the first theme. There were 4 categories about physical and cultural food access with 6 subcategories in the second theme, drawn from 47 codes including convenience stores, food delivery, food streets, temptation, media, and SNS. The third theme about drinking cultures in Korea consisted of 2 categories with 5 subcategories from 37 codes including alcohol drinking, social gatherings with alcohol, and typical foods with alcohol. The final theme was drawn from 38 codes including strong will, mandatory, challenge, patience and self-control which were categorized into 3 categories with 6 subcategories depicting burden of individual efforts on weight management.

Personally identifiable information was not collected. We assigned case numbers randomly during data collection and analysis to protect the anonymity of the participants. All research processes were approved by the IRB at Seoul National University in Korea (IRB No. 1400/001-012).

## 3. Results

The participants regarded weight management as a “must do,” since it contributed to their health, physically, mentally, and socially. In their busy lives in the city, convenience served as a major standard and condition for choosing behavioral options to manage weight (Topic 1). The built environment of Seoul, such as parks, walkways, and public transportation, were perceived to be convenient for physical activity, thus facilitating weight management. The increased convenience of the city’s food environment, however, challenged the participants’ efforts regarding weight management by offering greater availability of and accessibility to processed, prepared, cooked, or delivered foods rather than fresh meals (Topic 2). In addition to the physical environment, the sociocultural environment of Seoul was also said to influence weight management unfavorably with the culture of drinking alcohol with high-calorie foods (Topic 3). The participants recognized the influence of the food environment on their weight management, but they believed the environment was difficult to change or improve. Therefore, they felt a heavy burden at the individual level that they were responsible for managing their own weight (Topic 4). The following sections illustrate the results further under four overarching themes.

### 3.1. Convenience Comes First

**Figure 1:**
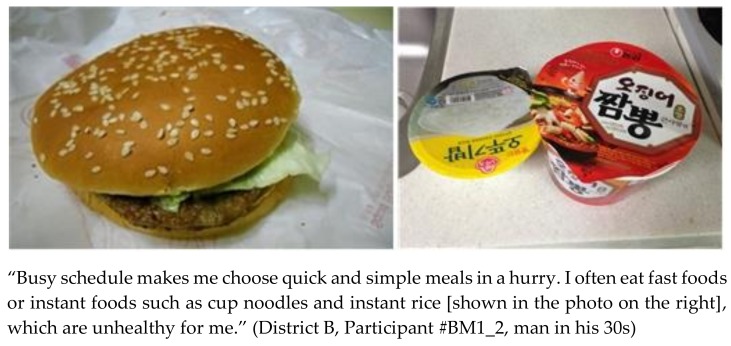
Pictures and statement depicting quick and simple meals.

The participants emphasized that weight management was something they had to make efforts toward every day. They valued achieving and maintaining a normal, healthy weight since it contributed to their self-esteem, self-satisfaction, confidence in relationship building, and health. They agreed that the healthiest dietary choice for weight management was “cooking and eating at home” with fresh groceries. However, this was impractical for them. In their “hectic daily lives,” wherein they had little time to relax, the participants regarded cooking as inconvenient and time consuming. Regardless of gender, age, occupation, and marital status, participants preferred “convenient” (i.e., simple and quick) ways of eating or meal types to healthy diets conducive to weight management (Figure 1).

Acting quickly and being in haste were perceived as part of the general social mood in Seoul, and participants complained of always feeling pressed for time. During their daily lives, they mostly did not want to spend time or energy in the complicated processes involved in preparing meals, such as purchasing, cooking, eating, cleaning, and disposing of food, all of which they believed to be time consuming. Meanwhile, their increasing options for meals allowed them to choose meals that suited their tastes at reasonable prices.

Participants’ busy city lives were also characterized by overtime work and less time for relaxation, both of which were accompanied by an irregular and unbalanced diet. Lunch was particularly quick and simple, often eaten at desks or in transit. Convenience stores had places to eat quick meals while standing, which was very natural and familiar to people from Seoul.

“With little time in between things, I eat on my way small bites of convenient food.”

(District B, Participant #BF3_1, woman in her 20s)

“I think I have purchased lunch and dinner most of the days since middle school.”

(District B, Participant #BF3_2, woman in her 20s)

“I often bring quick meals to the office when I am busy, mostly from convenience stores or fast food joints. Just like that. I think I do this 3 or 4 days a week.”

(District A, Participant #AM1_1, man in his 30s)

The same tendencies were found for dinners or meals at home. Seoul is well-populated with young residents living alone. A majority of young participants said they usually had small bites or nosh instead of full meals, relying on food from outside the home. In particular, groceries sold in large bundles were often impractical because they resulted in leftovers, so people preferred purchasing prepared meals. Preferred options included boxed meals from convenience stores, delivered food, and fast food. These convenience-seeking dietary behaviors were more prominent among men, who are not used to preparing meals for themselves in Korean culture.

“For me, home is just a place to sleep. I spend most of my time in my workroom, and eat frozen foods from convenience stores a lot.”

(District B, Participant #BF4_2, woman in her 30s)

“If you live alone and apart from your family, you should prepare your own meals, which is too much work for me. Therefore, I always have an irregular diet with unhealthy foods. Furthermore, my friends often visit me, bringing calorie-rich foods, so I tend to eat such foods often.”

(District A, Participant #AM1_2, man in his 20s)

“I do think the best practice is to prepare meals myself. But it’s too much of a hassle to actually cook. They sell groceries in big packages. I don’t get to use them all, and I end up throwing away the unused items. It’s a waste of food, you know. So, there are more days that I don’t cook at home. I want more stores to sell groceries in smaller packages.”

(District B, Participant #BM3_1, man in his 30s)

Female participants who lived with their partners or children were generally tasked with preparing meals for their family. Regardless of whether they had a job, the women assumed most of the housework burden. This made it a struggle for them to prepare meals for the family on time, and they found it time consuming and inconvenient. They had to prepare meals and snacks several times a day for each family member, each of whom had a unique schedule, and they tended to overeat in order to avoid leftovers. They preferred, therefore, dining out or precooked foods. These behaviors, evidently led by their preferences, were strongly contradictory to their own perception that homemade meals were best for weight management.

“When I prepare snacks or meals for children, I usually eat them together and even finish the food left by the children. I often overeat to finish it up [laugh].”

(District A, Participant #AF3_1, woman in her 40s)

“I have to give up convenience to stay healthy. Weight management should be a part of my daily life. I believe that the inconvenient and uncomfortable routines can give me what I want.”

(District A, Participant #AF6_6, woman in her 40s)

### 3.2. Tempting Food Environment

While convenience was an important criterion for participants’ food choices, the problem was that such foods were too available and accessible for them in the city. The availability and accessibility of diverse food outlets are relatively greater in Seoul than in other Korean cities due to its high density and mixed-use areas. Food choices have increased continuously over the years, including rapid increases in the availability of convenience stores and delivery services. This increasing trend has accelerated in part due to media influence.

Being offered a variety of food options in close physical proximity around the clock, participants were often enticed to eat beyond their regular daily meals; in other words, the close and constant exposure to food induced people to eat whenever they felt like it. This highly enticing food environment made participants think about eating frequently. They were enticed to eat “late-night meals” or snacks, which they might not have otherwise eaten or thought of. They believed that many of the foods available for nighttime snacks or snacks outside of regular meals (which were mostly from convenience stores or delivery services) were unhealthy because of being rich in fat, sodium, and calories. Those foods were, however, so stimulating that they became a considerable barrier for participants’ weight management.

“Almost anything is available for 24 hours in this district.”

(District B, Participant #BM1_2, man in his 30s)

“No one will be obese if we all live in the mountains. [Participants laugh.] No delivery of chicken. No pizzas.”

(District A, Participant #AF4_2, woman in her 40s)

**Figure 2:**
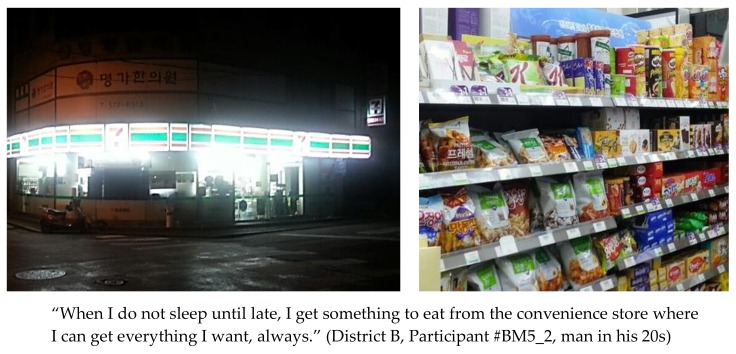
Pictures and statement depicting a convenience store and its products.

**Figure 3:**
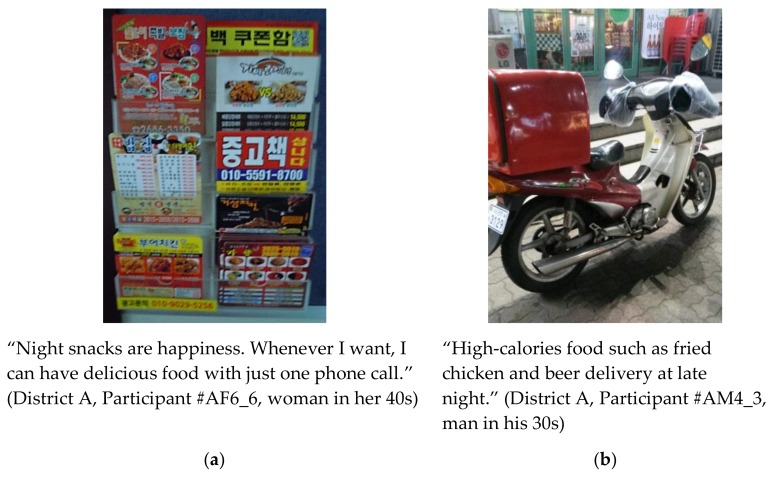
(**a**) Picture and statement depicting flyers and coupons for food delivery; (**b**) Picture and statement depicting a motorcycle delivering fried chicken.

Convenience stores in Seoul are located in residential areas as well as business districts, selling everything from finger foods to traditional Korean-style rice-and-soup sets 24/7 (Figure 2). Moreover, nearly every type of food can be ordered by phone or delivery service applications on mobile devices, delivering food to customers’ doorsteps or anywhere in Seoul, even in outdoor areas such as parks, until late at night or any time of day (Figure 3).

**Figure 4:**
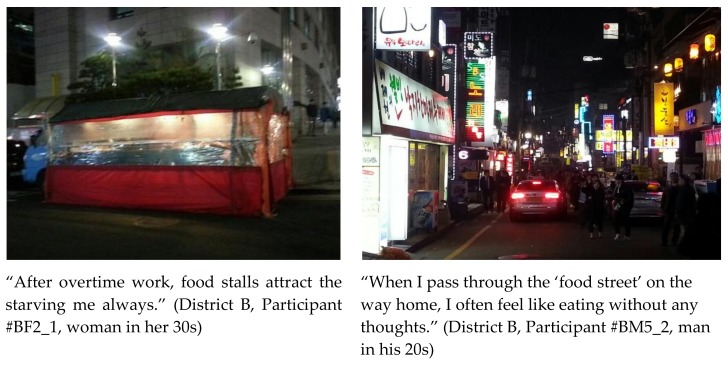
Pictures and statements depicting a food stall and a food street.

**Figure 5:**
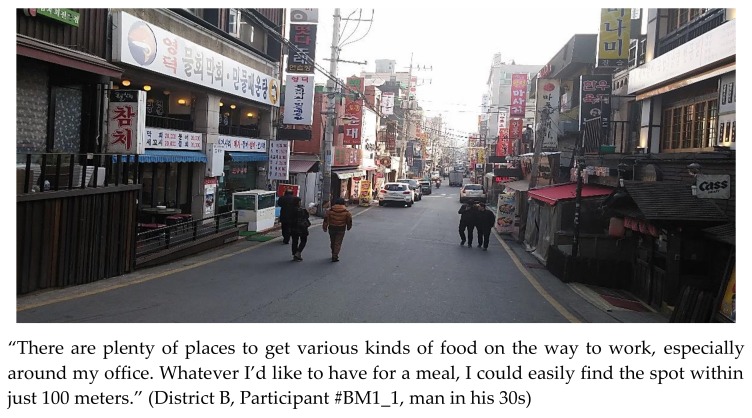
Picture and statement depicting various eateries along a road.

Restaurants, takeout vendors, grocers in varying sizes, and bars are also located near both residential and business areas in Seoul. Street food stalls attract commuters and pedestrians with high-calorie junk foods, especially at night (Figure 4). Most respondents commuted by public transportation and walked more to avoid heavy traffic jams. As they walked along the streets to and from bus stops and subway stations, they were “always” lured by the sights and smells of various types of food (Figure 5).

“By the time I leave work for home, I get very hungry. And there are many food stalls on my way home. I yield to the beautiful smell of foods and end up bringing them home. Then I eat them as night snacks. My weight management is challenged this way.”

(District A, Participant #AM3_3, man in his 30s)

Constant exposure to food information and images via media also stimulated participants’ appetites and enticed them to order late-night snacks that they had not intended to eat or had given up eating in the past when late-night delivery was not available. TV programs on food and dining have increased recently in great volume in Korea, many of which feature celebrities visiting and dining at famous and unique restaurants in the city. Moreover, there has been a proliferation of TV cooking entertainment programs, which frequently show overeating and appeal to viewers’ appetites. TV programs focused on eating (called *mokbang* in Korean, which translates to “TV programs on eating”) and cooking (called *cookbang* in Korean, which translates to “TV programs on cooking”) are mostly broadcast at night, usually after dinner. While watching such programs, participants could order what they wanted to eat, whenever and wherever, via delivery services. The participants agreed that they encountered “food porn” whenever they turned on their TVs.

“There is a subconscious desire to eat something delicious. So, when it is touched by something or someone, the subconscious awakens. [chuckles] I think I get weak and swayed by the temptation [of the food programs on TV].”

(District B, Participant #BF3_1, woman in her 20s)

“When I watch TV dramas, I want to eat in the restaurant where the main characters always get together.”

(District A, Participant #AF4_3, woman in her 30s)

Information about city dining is also widely shared on social media, including posts with various food-related hashtags (e.g., #eatstagram). Restaurants often use social network services (SNS) as their promotion channels. SNS users, for example, can get discounts when they share restaurant information on SNS. Such information is often accompanied by eye-catching, decorative, and appetizing photos of foods and restaurants, thus attracting people (particularly young women) to react to it. Participants also expressed that they wanted to do what others did and not fall behind on the trends. Trying to follow these trends, however, counteracted their weight management.

“Restaurant pages and food reviews are on SNS these days. Since many people participate in those postings, I get interested in them, and check them more often. The food featured on such online pages looks good and delicious, and people visit those restaurants and shops. It’s a trendy thing.”

(District B, Participant #BF3_3, woman in her 20s)

“On SNS, there are lots of advertisements of imported desserts at department stores, especially targeting young female customers. These desserts are pretty, unique, and special—you want to taste them and have them. It’s a great temptation.”

(District B, Participant #BF3_2, woman in her 20s)

### 3.3. Alcohol and Anju

**Figure 6:**
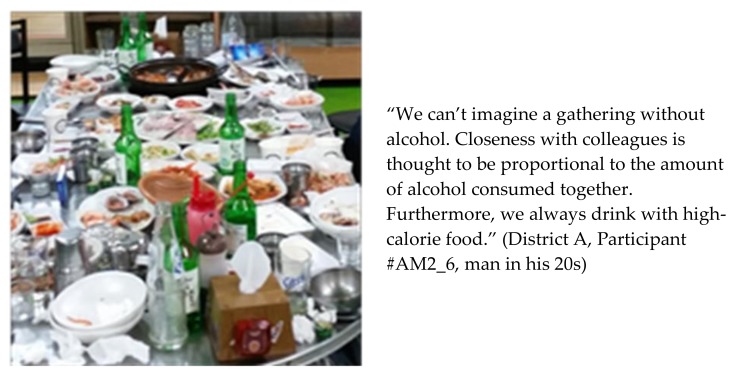
Picture and statement depicting a table with food and alcohol.

Dining out in groups and late-night snacking were usually accompanied by drinking alcohol. Quick lunches were also compensated by a heavy dinner with alcohol, in part as a way of releasing stress. According to the participants, alcohol is involved at gatherings with family, friends, colleagues, and business partners “by default” (Figure 6). Drinking alcohol is regarded as a way to socialize, break the ice, become friendly, change moods, and facilitate conversations; thus, it was recognized as part of their social lives. It was also common among participants to relax with alcohol at the end of a day. A can of beer after work or at home in the evening was considered a great relief and stress releaser.

The Korean term *anju* refers to foods paired with alcohol. The majority of participants agreed that drinking alcohol in restaurants and at the bar or at home was accompanied by eating *anju*. *Anju* consists mostly of greasy, fatty, or salty foods. For example, “*chi-mac*,” a shortened term in Korean for fried or barbequed chicken and beer, and “*sam-gyeop-sal* and *so-ju,*” Korean words for pork belly and distilled Korean liquor, are typical and popular pairings of *anju* and alcohol. Indeed, *chi-mac* was the most frequently mentioned delivery food. Although drinking alcohol and eating *anju* were perceived as highly prevalent in Korea, participants evaluated drinking alcohol, particularly the consumption of obesogenic foods inherent to it, as a barrier to their weight management.

“I check my weight everyday.… [By so doing] I learned that it’s not very good to repeatedly gain weight by drinking alcohol till late at night and dropping the weight by working out the next day.… We need a cultural change. A social gathering always involves alcohol. It starts with alcohol and ends with alcohol, always. We even drink after working out together [laughs].”

(District A, Participant #AM3_1, man in his 40s)

### 3.4. Burden of Individual Effort to Manage Weight

Most participants recognized the influence of urban environmental conditions that interfered with their weight management. Nevertheless, they believed that current urban environmental characteristics, both physical and sociocultural, are part of the era, making these characteristics difficult to change. Weight management, involving the practice of a healthy diet and physical activity, was, therefore, a matter of individual effort and responsibility. Between diet and physical activity, participants gave priority to diet, with the belief that controlling food intake would bring weight loss and visible changes in body shape much faster.

**Figure 7:**
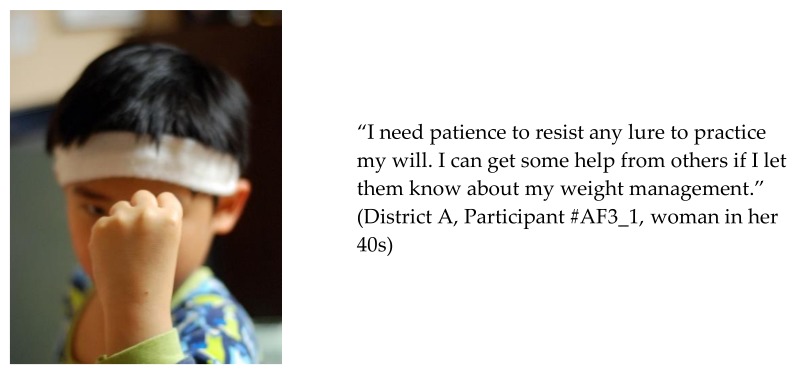
Picture and statement depicting an individual’s desire to practice his/her will.

The participants felt burdened by the need to overcome the temptations of the urban environment and practice healthy eating as well as other healthy behaviors through their own effort in everyday life. They felt “guilty” for having difficulties in practicing healthy behaviors, especially healthy eating in the current urban environment, and attributed successful weight management to an individual’s “strong will,” “motivation,” and “persistence” (Figure 7). Social support from personal social networks and financial resources were mentioned as facilitators of individual efforts.

“Will matters, really. Strong will. Persistence. I can drop lots of weight if I skip dinner and refrain from alcohol. Otherwise I gain weight. If I’m really determined not to drink, I can lose weight. I think I know how to lose weight. All that matters here is whether I have the will.”

(District A, Participant #AM2_6, man in his 40s)

“With the will and people to go together, I think [weight control] is possible. The chance is slim when I try alone.”

(District A, Participant #AM3_1, woman in his 30s)

## 4. Discussion

We examined the characteristics and influences of the perceived urban environment on weight management in Seoul using qualitative research methods. Participants emphasized eating convenience and preferred prepared meals that helped them save time and trouble. They had various food options with very few restrictions on availability (in terms of both time and place), and such an environment led to high availability of and exposure to obesogenic food. The culture of pairing alcohol and food also made it difficult for them to control their diets for weight management. Although they recognized the significant influence of physical and social environments, they felt pressured by a need to focus on individual efforts to manage their weight rather than anticipating or demanding environmental changes.

Previous studies on food environments, obesity prevention, and weight management have mostly focused on the accessibility and availability of food in terms of the density or proximity of food retailers [5,8,9,10,49]. Although their findings were not always consistent, researchers have found that quality of diet is higher with greater accessibility to fresh food outlets and lower with greater proximity of convenience stores [12,14,50]. Caspi et al. [51] pointed out the importance of considering diverse dimensions of the food environment such as availability, accessibility, affordability, accommodation, and acceptability. Aside from the type and location of food outlets in the community, the availability of healthy food in daily settings and the information environment, including media and advertising, [52] can affect dietary lifestyles.

Our study adds new findings related to the food environment and weight management in urban environments. The participants emphasized the influence of the perceived environment as well as physical environmental factors. They addressed their eating behaviors and weight management in the context of their hectic urban lives. Despite the high physical accessibility and diversity of food, they prioritized quick, simple, and convenient meals rather than healthy ones. The physical and cultural environments, with their highly tempting foods, were both perceived, regardless of time and place, as obesogenic, posing a challenge to attaining a healthy dietary lifestyle.

Most studies so far have addressed the food environments in cities in Western countries, which have rather different urban health contexts compared to Asian cities. In previous studies, food access referred to the availability of healthy choices, especially access to fresh food (in contrast to food deserts). Researchers have also examined the influences of urban food environments containing many obesogenic food outlets (e.g., fast food and convenience stores)—called “food swamps”—on obesity [11,53]. Improving access to healthy foods has been recommended as a way to prevent obesity through increasing healthy eating, but we must consider a different approach in terms of availability and accessibility in Seoul. Indeed, this qualitative study revealed that the perceived availability and accessibility of various food retailers was exceedingly high in Seoul. There were 10.3 convenience stores and 19.1 supermarkets per square kilometer on average in Seoul [54], which was more than 25% of the total number in Korea. In fact, the number of convenience stores and supermarkets in both Districts A and B in this study were greater than the average number of convenience stores and supermarkets in Seoul. This food environment enabled participants to get any type of food, including fresh food and fast food, in their neighborhood.

With the growing number of single households and small-size families in Seoul, people increasingly prefer smaller food portion sizes within close range of their homes [55]. In addition, sales of food delivery and takeout have been rapidly rising [56]. In 2015, compared to the previous year, the sales of packed meals from convenience stores in Seoul increased by more than 50% [11]. With the introduction of mobile applications for food delivery services, the number of users has increased rapidly, occupying about 40% of the market share in 2015. The development of delivery businesses in Seoul has made it possible to deliver both fresh and cooked foods: most grocery markets now provide delivery services, and e-commerce companies have begun fresh food delivery services, which has boosted fresh food sales and deliveries [57]. Despite the high availability and accessibility of both fresh and prepared food, the Seoul residents in this study seemed to be more likely to choose the latter. This was because they generally felt pressed for time and perceived themselves, and urban life, as always busy. In other words, their food choices mostly derived from their lifestyle characteristics and eating preferences, which favored convenience and speed. In fact, while food delivery and takeout have diversified in terms of types of food and hours of delivery, the number one delivered food in South Korea in 2015 was fried chicken, and the number one type of takeout food was fast food [56]. Recently, however, there has been increased production of fresh foods that are trimmed for quick and easy cooking and are put in small packaging appropriate for a single meal. These products are becoming popular alternatives for both healthy eating and convenience. Thus, it is necessary to monitor the effects of such changes on dietary lifestyles and obesity prevention [58].

Previous research on the food environment paid more attention to residential areas, underestimating the influence of the food environment at work and commuting routes despite the greater density of food retailers on these routes [4,49]. The results of this study reflect the high and constant exposure to diverse food outlets on Seoul’s commuting routes, in addition to the factors present in their residential areas. Because public transportation utilization is high in Seoul, commuters have more exposure to food outlets on their commuting routes, which in turn influences their weight management efforts. They feel strongly tempted by their constant exposure to various foods on their daily commuting routes. Especially when it was late at night after a period of overtime work, the temptation of food stalls or convenience stores posed a major challenge to weight management for many participants.

The effects of the high availability and accessibility of foods in Seoul were also linked to participants’ constant exposure to food in the media. The number and type of TV programs on eating and cooking (*mokbang* and *cookbang*, respectively) have increased since 2010, given their growing popularity among the public. In 2015, at least one or two new programs on cooking and eating debuted weekly on multiple TV channels in Korea [59]. Moreover, eating has been portrayed as a recreational activity or stress releaser rather than as a health behavior [60]. Such TV programs are viewed as a kind of “vicarious gluttony” for those who are pressured by weight control and as a way of eating with company for those who eat alone without their own cooking [61,62]. *Mokbang*, in particular, is now also common and popular on Internet Protocol TV, YouTube, and other SNSs in Korea. The cultural trends portraying eating as attractive and something to take pride in have accelerated people’s exposure to food and eating, alongside the development of information technology and the diversification of media.

Therefore, people’s high exposure to food with no limitations on time and place was perceived as one of the biggest barriers for all participants to obesity prevention. Seoul’s urban environment appeared also to be constantly finding ways to increase this exposure. Participants also felt that eating obesogenic foods, rather than healthy foods, helped them relieve stress in their daily lives. While participants believed they mostly knew what efforts were required to effectively manage their health and weight, they recognized that environmental factors needed to change to make those environments more supportive of such efforts.

Besides the physical environment, the eating culture was a factor that participants found difficult to change through their own efforts. For example, drinking alcohol in Korean culture was considered an exceedingly important factor for participants’ social lives, suggesting that it needs to be addressed through cultural changes. People can drink alcohol at most restaurants, even ones that are not bars, and can legally have alcohol delivered along with ordered foods [63]. In a culture where drinking alcohol is enjoyable, highly accessible, and highly available (there are few restrictions on time and place), people are naturally going to drink more. All participants regarded Korean drinking culture, especially the involvement of calorie-dense foods such as *anju,* as a major barrier to their weight management. Importantly, rather than expecting environmental changes (in terms of both their physical and cultural environments), participants felt that their own efforts to change and control their behaviors were the most important factors for weight management.

Previous studies focused primarily on the physical aspects of the local food environment, neglecting the fact that the food environment varies with the setting and culture. Quantitative approaches have been the main research strategy to investigate the relationships between food environment and eating behaviors and weight management. Such approaches are, however, limited in providing contextual understanding. The qualitative approach in this study, by contrast, helped to explore relationships in the specific context of Seoul. Previous qualitative studies on food environments and eating behaviors mostly addressed individual preferences, social networks, and physical environments as the factors influencing diet [64,65,66,67,68]. In addition to those factors, we found that cultural context was very important for understanding influences. The effect of physical accessibility and availability might also act as both a facilitator and a barrier, depending on the cultural characteristics of an urban setting. Therefore, different contextual understandings according to the unique environments of Seoul’s urban food environment are meaningful for suggesting a favorable environment for weight management.

This study had a number of limitations. First, participants were all in their 20s to 40s and voluntarily participated in the photo elicitation interview process (i.e., taking digital photographs and writing comments about the photographs electronically, sending those data via email, and participating in the group discussions twice). This data collection process might have limited the participation of individuals with poor computer or digital literacy and/or mobility. Second, although all participants had been diagnosed with obesity or as overweight within two years prior to the study, their individual experiences with weight could differ by level of obesity. According to the IRB, we could not ask for participants’ weight or BMI in this study. We focused on exploring shared perceptions and experience of obesity and weight management in Seoul rather than on investigating the differences in experience by obesity level. Although we do not have information about how obese the participants were in the past, it is notable that none of the participants admitted any experience of discrimination or stigma related to obesity. Third, although socioeconomic status is known to be an important factor influencing obesity, we stratified the participants only by age and gender. Following the advice of the IRB, we did not collect participants’ socioeconomic status since it could be considered sensitive information. We did, however, consider employment and family status during recruitment, factors that affect lifestyles choices in daily life. Despite these limitations, this qualitative inquiry still helps us understand participants’ experiences with and perceptions of the unique urban environments associated with weight management in Seoul.

While more research and critical discussions are warranted on the relationships between obesity and urban built environments in Asia from diverse perspectives, this study is one of the few, to our knowledge, to expand the discussion on obesogenic environments in a single Asian city. Our findings suggest how the urban environment interacts with eating behavior and weight management in a certain context, offering unique variations on or contrasts with existing accounts. While participants were selected from two districts with different prevalences of obesity, their perceptions of the food environment were found to be similar. As such, despite the differing environments, there were no major differences in perceptions of the food environment in terms of weight management. Further research is needed that considers both actual and perceived environments to address the factors influencing weight management.

In South Korea, weight consciousness is currently very high [23]. However, as shown in this study, the food environment in Seoul is perceived as a major barrier to weight management efforts. There have been recent rapid changes in the population structure of South Korea, including the low birth rate, rapid aging, and an increased number of single households. These social changes have deepened the culture of preference for and habituation to quicker and easier lifestyles in both individuals and environments, producing barriers to the practice of healthy behaviors and obesity prevention. It is important to emphasize the roles and responsibilities of the governmental, academic, and commercial sectors for weight control, beyond those of individuals. From a public health perspective, environmental changes are needed to make it possible for people to choose healthy food options without complex and time-consuming cooking processes, catering to the priority in Seoul for convenience and speed. To relieve the burden on individuals to manage weight and to facilitate the practice of healthy behaviors, legislation to regulate overexposure to obesogenic foods in terms of time and place can be suggested. This calls for intersectoral communication and collaboration to promote healthy eating and healthy weight management as well as a supportive urban environment. Such interdisciplinary collaboration is also warranted to address the complexity of the relationship between obesity and the urban environment through innovative (both conceptually and methodologically) research.

## 5. Conclusions

Employing the photo elicitation interviews, this study explored the context of the urban food environment as it relates to weight management among residents of Seoul, Korea. We identified four themes through thematic analysis: (1) convenience comes first, (2) enticing food environment, (3) alcohol and *anju*, and (4) burden of individual effort to manage weight. The participants preferred convenience in their eating, which meant quick and simple ways to eat. Constant exposure and close proximity to food, both physically and culturally, were identified as the most salient characteristics of the urban food environment in Seoul. Alcohol was accessible in most food venues, from mobile food stalls to food delivery, and alcohol drinking, which is often accompanied by high-calorie foods, was a major challenge to weight management.

Though they were influenced by their physical and cultural environments, the study participants tended to perceive weight management as a matter of individual will and effort. They believed that environmental and social changes are difficult to make, especially in an era of economic challenges, when most food vendors are small independent businesses trying to make a living.

The findings of this study illustrate an aspect of the current food environment in a megacity and emphasize context-based approaches to urban health. A systematic change toward a more supportive environment for healthy eating and weight management is recommended.
